# Determination of aluminum and zinc in infusion tea cultivated in north of Iran

**DOI:** 10.1186/s40201-015-0196-9

**Published:** 2015-05-31

**Authors:** Mahboobeh Ghoochani, Sakine Shekoohiyan, Masoud Yunesian, Shahrokh Nazmara, Amir Hossein Mahvi

**Affiliations:** Department of Environmental Health Engineering, School of Public Health, Tehran University of Medical Sciences, Tehran, Iran; Department of Environmental Health Engineering, School of Public Health, Hormozgan University of Medical Sciences, Bandar- Abbas, Iran; Center for Solid Waste Research, Institute for Environmental Research, Tehran University of Medical Sciences, Tehran, Iran; National Institute of Health Research, Tehran University of Medical Sciences, Tehran, Iran

**Keywords:** Black tea, Aluminum, Zinc, Iran

## Abstract

To determine aluminum and zinc levels in black tea cultivated in north of Iran, 105 black tea samples were collected from the tea growing regions of Guilan and Mazandaran provinces and were analyzed for Al and Zn concentration of tea infusion. Contents of all elements were analyzed three times separately by using an Inductively Coupled Plasma Atomic Emission Spectrometry (ICP - AES). The solubility of Al and Zn in infusions at 5, 15 and 60 min with boiling water showed that the mean level of Al in the third infusion was the highest (262.09 mg/kg) and in the first infusion was the lowest (169.40 mg/kg). The mean level of Zn in the third infusion was the highest (51.40 mg/kg) and in the second infusion was the lowest (48.33 mg/kg). The analysis of results also showed that the location factor influences the contents of these metals at different infusions.

## Introduction

Tea is one of the oldest, most popular and non-alcholic drinks in the world and it is provided from the dried leaves of the tea plant [1]. Economic and social benefits in tea are clear from the fact that about 18–20 billion tea cups are consumed daily in the world. Black and green teas are the popular version [2]. Iranians keep one of the highest per capita rates of tea consumption in the world, (about1.6 kg per capita consumption in the period from 2005 to 2007 [3]. Approximately 34 thousand hectares of lands have been cultured for tea in Guilan and Mazandaran provinces, Guilan and Mazandaran have a humid temperate climate with enough annual precipitation. Almost half of the dry tea interior production and the rest come from imports [4]. Many researchers have showed that tea has some useful health effects, including the prevention of diseases such as Parkinson, skin cancer, myocardial infarction, and coronary artery diseases [5]. Tea is exceptionally rich in polyphenolic compounds (flavonoid and phenolic acid), which are powerful antioxidants that protect the body against disease [6] and contains minerals and trace elements that are essential to human health. The determination of total elements content in different beverages has been a subject of numerous studies. On average, 1 liter of tea is consumed per person per day in Iran and UK which as a percentage of average daily dietary intakes, can provide 58.8 % of Al, 0.44 % of Zn and 58.8 % of Al, 2 % of Cu and 115 % of Mn [7, 8]. Studies by Wang, Su, and (1994) show that of the daily dietary intake of 9–12 mg Al by Chinese population, tea contributes 0.2–1.1 mg Al, assuming that an adult drinks 1–5 g of tea per day. The Provisional Tolerable Weekly Intake (PTWI) for Al of 7 mg/kg body weight (equivalent to 1 mg/kg body weight/day) was considered by the Joint FAO/WHO Expert Committee on Food Additives (JECFA [9]). Al was affirmed as a food contaminant in 1989. Various studies have been conducted, to evaluate the daily dietary intake of Al from a number of food products. Studies on dietary intake of Al show an average intake of 23 mg/day for Indian population [10], 9– 12 mg/day for Chinese population [11], 2–25 mg/day for American population, 2.2–8.1 mg/day for Japanese adult males and 0.6–33.3 mg/day for Dutch adults [12]. The contribution of tea drinking to mineral absorption is not certain, as the bioavailability of many of these metals with tea is not known [13]. Jackson reports that tea which contains a substantial amount of Al may present health hazards (kidney weakness) for consumers [14]. Moreover, high Al content in the human body has been hypothesized to have possible links with various diseases, such as encephalopathy dementia, oestomalacia, fractures and high levels of bone Al and Alzheimer’s disease [15–17]. Although tea leaves and leaf infusions contain high concentrations of Al, only a small proportion of it is available for absorption in the gastro-intestinal tract [13, 18] and the renal excretion of Al is fairly effective [19]. It has been observed that tea, ingested alongside food, inhibits the absorption of inorganic and some forms of organic iron, contributing to iron deficiency, mainly in women on a vegetarian diet of low iron content. Studies show that tea drinking may marginally decrease the availability of divalent metals, such as Cu and Zn [13]. The Zn is known to be essential micronutrients, but can be toxic depending upon the concentration. The low or high amount of dietary intake of Zn was based to create the various physiological and ethological diseases because of these trace element deficiency or toxicity [20].

The origin of Iranian tea is from three varieties of seed from the Northern part of India (from the Assam region of India) [21]. Most of the farms are located the hillsides of Iran like the farms in Darjeeling. These farms produce an orthodox style of black tea. The color of Iranian tea is red with fairly light taste, and it is delicious without adding any milk or sugar. Institute of Standards and Industrial Research of Iran has set the maximum permissible levels of elements in the black tea only for As, Hg, Pb, Cd and Cu, being less than 1, 0.05, 1, 0.1 and 50 μg/g, respectively [22]. In addition, black tea has high amount of fluoride which can be released in tea liquor as well [23]. Also, it should be noted that the heavy metals migration to plants can be as a matter of fertilizers application [24]. Tea is an indispensable part of everyday life for many people in Iran, so we decided to do this study to ensure that public health is maintained. In the present study, tea samples were collected from different parts of Guilan and Mazandaran provinces to determine their aluminum and zinc contents.

## Materials and methods

**Fig. 1:**
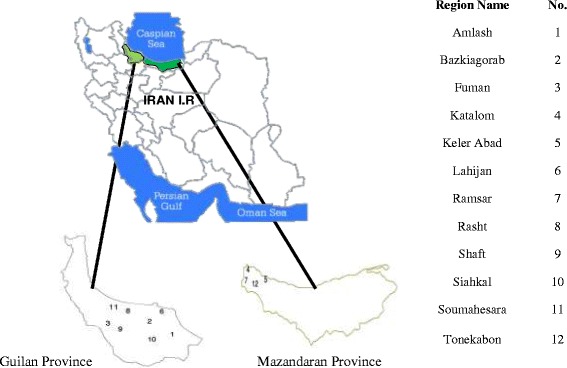
Location of sampling regions in Guilan and Mazandaran provinces

This research was done between September and November 2010 on tea samples that are cultivated in Guilan and Mazandaran Provinces in north of Iran (Fig. 1). 105 black tea samples were selected randomly from 105 farms in the two different regions of Mazandaran and Guilan provinces.

The weight of each sample was about 100 g. The glassware containers used for analysis were washed with detergent and rinsed several times with tap water to remove absorbance due to detergent; then they were soaked overnight in 6 N HNO_3_ (Merck) solutions and finally rinsed with deionized water. All aqueous solutions and dilutions were prepared with ultrapure water (c). 5 g of each tea sample was weighted by a digital analytical balance (Mettler Toledo, Switzerland) with ± 0.0001 g precision and then was added to 500 mL of boiling tap water and allowed to infuse for 5, 15 and 60 min. Then samples were filtered under vacuum (using a Whatman No.42 filter paper) to eliminate any turbidity or suspended substance. Liquors from the first, second and third infusions were analyzed for aluminum and zinc contents by Inductively Coupled Plasma Optic Emission Spectrometry End of Plasma (ICP-OES EOP, Spectroacros, Germany). The blank solution was prepared in similar way without black tea. The purity of argon as carrier gas was 99.999 % (grade 5), with a flow rate of 0.7 L/min for supplementary and Modified Lichte nebulizer and 13 L/min for coolant flow. The speed of 4 channel peristaltic pump was 60 rpm for 45 S in pre-flush condition and 30 rpm for analysis. The power level was adjusted on 1400 KW. Before quantitative analysis of samples, calibration curves of the desired metals were prepared using a series of diluted standard solutions. The recovery percentage, detection limits, and % R.S.D for triplicate measurements of the measured elements were 90 %–95 %, 0.3 ppb, and less than 5 %, respectively. Statistical analysis of the obtained results was performed by SPSS 18 and One Way ANOVA test.

## Results

The results of Al and Zn obtained from tea infusion samples in 5, 15 and 60 min are presented in Table 1. Aluminum levels were different from 0.00 to 1516.27 mg/ kg of dry weight (mean = 169.40 mg/ kg) for first infusion, 0.00 to 1851.66 mg/kg (mean = 223.52 mg/kg) for second infusion, 0.00 to 1446.83 mg/kg (mean = 262.09 mg/kg) for third infusion. Zinc levels were different from 0.00 to 113.66 mg/ kg of dry weight (mean = 49.55 mg/kg) for first infusion, 0.00 to 103.27 mg/kg (mean = 48.33 mg/kg) for second infusion, 0.00 to 141.10 mg/kg (mean = 51.40 mg/kg) for third infusion. One factor analysis of variance (ANOVA) of the data showed that levels of aluminum and zinc in different groups (infusions) were significantly different among the different black tea samples in different locations (*P* < 0.001). Table 2 presents the amount of Al content according to locations and infusion time. The maximum level of Al was determined in Amlash, which the amount of it in infusion time 5, 15 and 60 min were 633.44, 1020 and 982 mg/kg, respectively. The minimum level of Al was determined in Ketalam for first and second infusion and in Kelar Abad for third infusion, the amount in infusion times of 5, 15 and 60 min were 44.89, 54.08 and 49.39 mg/kg, respectively. Also results showed location influenced upon the amount of Al in infusion tea samples (*P* < 0.001). Table 3 showed the amount of Zn content according to locations and infusion time. The maximum level of Zn was determined in Kelar Abad for first and second infusion and in Lahijan for third infusion, the amount of this metal in infusion times of 5, 15 and 60 min were 77.64, 58.50 and 71.28 mg/kg, respectively. The minimum level of Zn was determined in Shaft for first infusion and in Soumahesara for second and third infusion, for which the amount of this metal in infusion times of 5, 15 and 60 min were 32.76, 33.47 and 32.66 mg/kg, respectively. Also results showed location influenced upon the amount of Zn in infusion tea samples (*P* < 0.001).

**Table 1:** The levels of aluminum and zinc in different infusions (n 630)

Parameters (mg/ kg)		Infusion Times(min)										
		5			15				60			
Min		Max	Mean	SD	Min	Max	Mean	SD	Min	Max	Mean	SD
Al	0.00	1516.27	169.40	220.0	0.00	1851.65	223.52	301.18	0.00	1446.83	262.09	294.86
Zn	0.00	113.66	49.55	21.98	0.00	103.27	48.33	19.49	0.00	141.10	51.40	26.96

**Table 2:** The levels of aluminum (mg/kg) in different infusions of tea samples (n 315)

Region	Number of sample	Infusion times (min)											
		5				15				60			
		Min	Max	Mean	SD	Min	Max	Mean	SD	Min	Max	Mean	SD
Amlash	30	182.65	1516.27	633.44	399.59	465.87	1851.65	1020.47	409.54	539.81	1446.83	982.80	284.57
Bazkiagorab	3	256.97	256.97	256.97	0.00	242.83	242.83	242.83	0.00	133.56	133.56	133.56	0.00
Fuman	24	16.39	223.73	104.30	84.17	16.25	235.08	106.92	86.51	14.64	189.73	82.51	67.25
Katalom	60	4.87	219.67	44.89	46.06	12.78	124.76	54.08	24.31	17.91	482.48	180.66	157.76
Keler Abad	18	3.45	211.60	84.48	86.26	0.00	194.43	69.57	75.21	0.00	138.30	49.39	55.49
Lahijan	45	53.79	256.24	154.79	66.33	84.20	278.33	171.09	57.02	72.17	676.27	308.82	154.08
Ramsar	3	46.37	46.37	46.37	0.00	78.90	78.90	78.90	0.00	288.84	288.84	288.84	0.00
Rasht	21	33.68	384.99	110.19	135.15	17.73	435.71	209.85	168.85	14.77	445.34	203.03	177.50
Shaft	12	62.67	197.84	135.44	55.77	93.63	281.79	209.17	83.37	80.00	282.11	192.37	83.67
Siahkal	48	45.23	541.36	215.92	152.22	0.00	645.84	250.67	212.58	0.00	663.40	216.69	198.12
Soumahesara	6	39.69	61.06	50.38	15.11	38.51	72.18	55.35	23.82	30.95	84.73	57.84	38.03
Tonekabon	45	0.00	354.10	87.07	125.67	0.00	457.37	143.35	161.30	0.00	474.04	152.92	172.29

**Table 3:** The levels of zinc (mg/kg) in different infusions of tea samples (n 315)

Region	Number of sample	Infusion times (min)											
		5				15				60			
		Min	Max	Mean	SD	Min	Max	Mean	SD	Min	Max	Mean	SD
Amlash	30	25.40	85.09	51.00	20.87	30.09	70.31	51.82	14.29	27.29	89.04	46.45	24.99
Bazkiagorab	3	44.31	44.31	44.31	0.00	45.50	45.50	45.50	0.00	36.19	36.19	36.19	0.00
Fuman	24	28.58	60.77	46.49	10.78	30.47	54.38	44.65	8.29	28.97	51.47	41.22	8.68
Katalom	60	17.43	84.49	38.86	14.72	29.67	102.27	46.02	15.35	24.94	141.10	63.82	34.04
Keler Abad	18	27.91	113.66	77.64	31.98	1.82	102.16	58.50	33.81	1.06	65.06	41.80	22.36
Lahijan	45	24.14	78.02	51.88	13.66	29.96	95.45	56.92	17.65	25.82	136.07	71.28	29.66
Ramsar	3	34.19	34.19	34.19	0.00	41.69	41.69	41.69	0.00	56.86	56.86	56.86	0.00
Rasht	21	20.79	44.04	36.81	5.66	26.29	42.87	35.80	6.46	26.49	41.99	35.38	6.57
Shaft	12	27.73	36.03	32.76	3.67	31.78	44.09	36.78	5.49	31.20	44.45	36.24	5.78
Siahkal	48	23.18	99.34	63.35	23.67	0.00	103.27	55.53	28.53	0.00	104.14	50.15	26.05
Soumahesara	6	35.71	36.73	36.22	0.72	29.67	37.27	33.47	5.37	23.10	42.21	32.66	13.52
Tonekabon	45	0.00	88.71	49.67	29.19	0.00	73.57	42.25	17.94	0.00	70.22	39.50	20.53

## Discussion

The results of study showed that the infusion time influences the contents of aluminum and zinc but their trend is different. The solubility of Al and Zn in infusions at 5, 15 and 60 min with boiling water showed that the transfer of Al to the brew was positively correlated with the infusion time. The mean level of Al in the third infusion was the highest. The mean level of Zn in first infusion, second and third infusion was 169.40, 223.52 and 262.09 mg/kg, respectively. Relationship between Zn and infusion time is a little different as in first and third infusion the metal levels were positively correlated with the infusion time but in second infusion the metal levels were negatively correlated with the infusion time. This different is very little. The mean level of Zn in first infusion, second and third infusion was 49.55, 48.33 and 51.40 mg/kg, respectively. Moghaddam et al. reported the solubility of Aluminum and Zinc in 31 Iranian consumed tea samples in the first infusion (2 min) was significantly higher than the second infusion (5 min) and the solubility in the second infusion was also significantly higher than the third infusion (10 min) [7]. Mehra et al. [8] reported the solubility of Al, Cu and Mn in infusions at 2, 5 and 10 min with boiling water. Their results showed that in the first infusion, the solubility was the highest and in the third infusion, the solubility was the lowest. The concentration of Al in the first, second and third infusion were 29.7, 10.4 and 3.3 mg/kg [8]. Currently, no national standard for Al and zn in in the black tea was established in Iran, only Institute of Standards and Industrial Research of Iran (ISIRI) has set maximum permissible levels of elements only for As, Hg, Pb, Cd. The study was done by Ebadi et al. [25] in Guilan province (Iran) on green leaf of tea cultivated in Lahijan and Fuman cities. The results showed the amount of Zn was below standard measure (10 ppm) and there was no need to be concerned about the amount of Zinc in tea samples [25]. In this research the Zn content in all infusion and in all location of Mazandaran and Guilan province is more than 10 ppm. Yemane et al. [1] reported the Zn levels in the clonal tea leaves samples were 67.9 mg/kg (1). Ashraf et al. [2] reported the Zn contents of 17 black tea sample were in the range of 23.7-122.4 mg/kg (mean = 65.7 mg/kg). The international comparison of Zn concentration was in Turkey (Narin et al.) 140.9 [26], Spain (Pedro et al. 2001) 43.2 [27], Japan (Matsuura et al.) 36.6 [28], China (Han and Li) 25.5 [29] and India (Naithani and Kakkar) 39.5 mg/kg [30]. Salahinejad and Aflaki [3] reported the mean of Al and Zn levels in Iranian black tea samples and tea infusion were 1.143, 449.3 and 24.10, 8.86 mg/kg, respectively [3]. The study of Olivier et al. [6] showed the concentration of Al and Zn in residue of infusion of eight traditional and herbal teas from different geographic regions. The mean concentration of Al and Zn in tea Africa, Ceylon, Oriental and America were 246.3, 344.4, 351.6, 66.9 mg/kg and 12.1, 15.1, 15.7 and 33.4 mg/kg, respectively [6]. The study of Ansari et al. [31] showed the Al and Zn levels in 30 samples of black tea cultivated in Iran were 699.2 and 40.3 mg/kg, respectively. The Al could be accumulated in tea leaves up to 23,000 mg/kg which considered much higher than the other plants accumulation which do not normally exceed 200 mg/kg. As the Al content of tea is dependent to its concentration in soil, applying best agricultural practice (BAP) would be considered as an alternative to control the Al contents in tea leaves.

Because of lack of sufficient information about the acceptable contents of Al and Zn in tea, it was decided to interpret our result with their allowable or acceptable daily intakes (ADI).

Table 4 represents the allowable or acceptable daily intake (ADI) of aluminum and zinc and reference or recommended daily intake (RDI) of them [32]. With 1.6 kg per capita consumption of black tea leaves annually, Iranian daily consumption averages 4.3835 g of black tea leaves per person [3]. Based on Table 4 the acceptable daily intake of aluminum is less than 50. According to average daily black tea consumption in Iran (4.3835 g per person) aluminum content in all samples was less than the acceptable daily intake. Salahinejad and Aflaki [3] reported the average daily intake of Al by consumption of black tea infusions was low rather than ADI [3]. However, it must be stated that tea drinking may contribute towards Al toxicity in individuals with impaired absorption or excretion of Al in their systems. Since the tea cultivation lands in north of Iran provide much of black tea being consumed in Iran and the exported amount to other countries, it is recommended all toxic elements and essential mineral elements in black tea samples and their infusions to be determined for safe consumption of black tea.

**Table 4:** Acceptable Daily Intake (ADI) of toxic metals and Recommended Daily Intake (RDI) of essential minerals for adults

Element	ADI (mgday-1)	RDI (mgday-1)
Al	<50	-
Zn	-	8-11

Since the consumption of tea in Iran is very high, beside regarding the probable release of heavy metals in the infusion tea, considering heavy metal free drinking water or treated with effective methods are suggested [33].

## Conclusions

Tea is one of the heavily consumed beverages in the world and on average in Iran one liter of tea is consumed by a person per day. In this regard health aspects related to tea is very important and therefore consumers should be very confident on the absence of any pollutants in black tea. In this study concentrations of aluminum and zinc were measured in black tea samples from different locations in different infusion time. The analysis of results showed that the location factor influences the contents of these metals at different infusions. Due to the lack of standards for all elements (toxic and essential mineral) in tea, it is recommended that the maximum allowable and safe concentrations to be established.
